# Personality disorder in autism spectrum disorder : myth or reality?

**DOI:** 10.1192/j.eurpsy.2023.2063

**Published:** 2023-07-19

**Authors:** N. Kouki, A. Maamri, S. Cherif, E. Cherif, H. Zalila

**Affiliations:** 1 Outpatient; 2 Child and adolescent psychiatry department, Razi hospital, Manouba, Tunisia

## Abstract

**Introduction:**

Autism spectrum disorder is a neurodevelopmental disorder characterized by persistent deficit in communication and social interaction associated with repetitive, restricted interests, behaviors or activities. Regardless long-term care, sequelae may remain present particularly in cognitive patterns, social interactions and adaptive reactions, leading to personality disorder in adulthood.

**Objectives:**

In this study we aimed to explore personnality disorder comorbid with autism spectrum disorder .

**Methods:**

Our study was based on the PubMed interface and adapted for 2 databases: Science Direct and Google Scholar using the following combination ( autism spectrum disorder [MeSH terms]) AND (personality disorder [MeSH terms]) covering the period from 2013 to 2022.

**Results:**

We initially reviewed 13 articles. At the end of the literature selection process, two articles were included.

The prevalence of personality disorders with ASD was estimated around 35%.

The personality disorders evoked mainly responded to cluster C associating an obsessive-compulsive and avoidant personality in respectively 32% and 25%.

Cluster A personality disorders, in particular schizoid personality, were found in 21% with a female.

Concerning cluster B, borderline personality disorder was the most frequent because of different symptoms overlapping . In fact, the prevalence of borderline personality disorder in ASD was 4% . Meanwhile the prevalence of ASD in borderline personality disorder was 3%.

**Conclusions:**

Apart from other neurodevelopmental pathologies, ASD can be comorbid with personality disorder. However, the neurocognitive particularities of ASD reveal clinical manifestations similar to those found in personality disorders. Therefore, additional research using large sample sizes and validated diagnostic tools taking into account the specificities of this population remain necessary.

**Disclosure of Interest:**

None Declared

